# Assessing Risk to Articular Cartilage and the Calcaneofibular Ligament During Fibular Nailing: A Cadaveric Study

**DOI:** 10.1177/10711007251351314

**Published:** 2025-07-20

**Authors:** Hirbod Abootalebi, William Mayer, Erin Bigney, Siyum Mohiuddin, Xiuming Shi, Madeline Power, Jacob Matz

**Affiliations:** 1 Dalhousie Medicine New Brunswick, Saint John, NB, Canada; 2 Canada East Foot & Ankle, Saint John, NB, Canada; 3 Faculty of Medicine, Department of Medical Neuroscience, Halifax, NS, Canada; 4 Horizon Health Network, Saint John Regional Hospital, Saint John, NB, Canada; 5 Canada East Spine Centre, Saint John, NB, Canada; 6 Saint John Orthopaedics, Saint John, NB, Canada

**Keywords:** Fibula, ankle fracture, intramedullary nailing, calcaneofibular ligament, soft tissue risk assessment, minimally invasive

## Abstract

**Background:**

Traditional surgical fixation of ankle fractures with plates and screws carries risks such as wound complications, hardware prominence, and soft tissue irritation. Intramedullary (IM) fibular nailing provides a minimally invasive alternative with potentially lower complication rates. Although prior studies have examined the risk posed by fibular nailing to the peroneal tendons and nerves, data remained limited regarding its impact on other adjacent structures especially the calcaneofibular ligament (CFL) and the articular cartilage of the distal fibula, structures whose injury could contribute to joint instability, persistent pain, or degenerative change.

**Methods:**

This study assessed the risk and extent of damage to anatomical structures during IM nail fixation on 10 cadaveric lower extremities. Risks were categorized based on distances from the nail to the CFL, anterior talofibular ligament (ATFL), sural nerve (SN), superficial peroneal nerve (SPN), peroneus longus, peroneus brevis (PB), and articular cartilage: high-risk (0-5 mm), moderate-risk (5.1-10 mm), or low-risk (>10 mm).

**Results:**

Macroscopic evaluation identified the CFL, ATFL, and PB as high-risk structures. The CFL was damaged in 3 of 10 specimens, ranging from 14% to 64% of its width. The average distances to the CFL (1.20 mm), ATFL (3.43 mm), PB (3.19 mm), and articular cartilage (3.45 mm) fell in the high-risk range, although no significant damage was observed to the articular cartilage, ATFL, SN, or SPN.

**Conclusion:**

This study further confirms that IM fibular nailing is a generally safe and reliable option for treating ankle fractures. However, attention should be given to the potential for iatrogenic damage to high-risk soft-tissue structures, particularly the CFL and peroneal tendons. Although cartilage was always spared in this cadaveric study, its proximity warrants surgical caution.

**Clinical Relevance:**

These findings clarify the soft tissue risks associated with IM nailing and may provide guidance for orthopaedic surgeons and patient discussions, emphasizing the importance of proper technique to preserve soft tissues.

## Graphical abstract

**Graphical Abstract:**
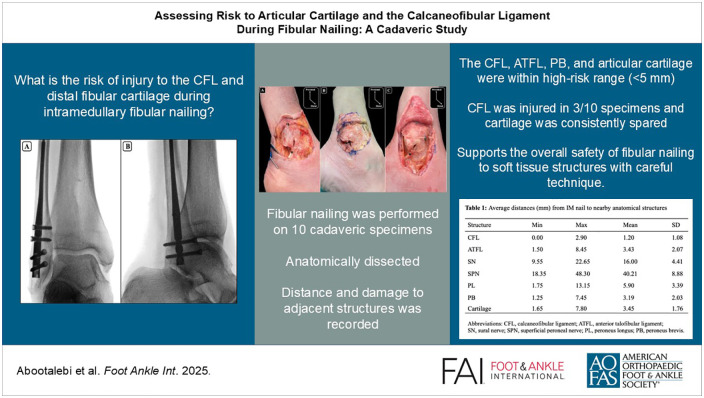
This is a visual representation of the abstract.

## Introduction

Plate and screw fixation has traditionally been used in ankle fractures requiring surgery. However, this method has drawbacks, including a risk of infection and wound healing complications, hardware prominence, and soft tissue irritation.^10,18^ In recent years, intramedullary (IM) fibular nail insertion has gained popularity as a minimally invasive option for ankle fracture fixation. Several clinical studies have assessed the efficacy of IM nail fixation, demonstrating its potential for decreased wound complications and diminished hardware prominence, making it a particularly attractive option for certain high-risk patient populations, such as those with soft tissue compromise, advanced age, diabetes, or immunosuppression.^1-4,19,20,25^ Both plate and screw constructs and intramedullary nails have a role in the fixation of lateral malleolar fractures and, ultimately, the fracture patterns and patient characteristics influence the construct of choice.

Previous studies evaluated the risk to adjacent structures during intramedullary fibular instrumentation.^9,14,17,23^ Iorio et al^14^ evaluated the potential risk of harm to the peroneal tendons and the sural nerve by assessing the different entry points in the fibula using 1.1- and 2.0-mm K-wires. Although they observed no injuries with both anteromedial and anterolateral placements, their study concluded that the anterolateral quadrant was associated with the lowest overall risk to these structures. Telgheder et al^23^ evaluated retrograde medullary screw insertion in 10 fresh frozen cadaveric lower extremities. Their measurements showed that structures such as the peroneal tendons, sural nerve, anterior talofibular ligament (ATFL), and calcaneofibular ligament (CFL) were maintained at safe distances with no iatrogenic injuries observed. Similarly, Medda et al^17^ placed a 3.5-mm cortical screw in 4 cadaveric specimens and noted that although the ATFL and CFL were closest to the screw head, no injuries were observed. In the only study evaluating fibular nails, Goss et al^9^ further examined risk to the sural nerve (SN), superficial peroneal nerve (SPN), peroneus brevis (PB), and peroneus longus (PL) tendons, identifying the PB as most at risk, with moderate to high risk also noted for the PL and SPN—yet no injuries occurred.

Despite these studies, there remains a scarcity of data regarding the risk of injury to other structures adjacent to the start point of the fibular nail, namely, the articular cartilage of the distal fibula in the lateral gutter and the CFL. Injury to articular cartilage can precipitate a cascade of events ultimately resulting in degenerative changes and arthritis, a consequence that may not be adequately captured by short-term studies.^13^ The CFL is essential for ankle and subtalar joint stability, especially during inversion and plantarflexion.^15^ Damage to the CFL can potentially lead to adverse outcomes, including compromised ankle stability and altered joint mechanics.^12,24^ This instability increases the risk of recurrent ankle sprains and may contribute to the development of chronic ankle instability.^16^

In this cadaveric investigation, we assessed the implications of fibular nailing on the articular cartilage in the lateral gutter of the ankle as well as the integrity of the CFL. Based on their proximity to the nail entry point, we hypothesized that both the articular cartilage and CFL would sustain damage during the procedure. Damage to other nearby structures was also documented.

## Methods

### Specimens

Ten human cadaveric specimens, spanning from the mid-tibia to the tip of the toes, were used. These included 6 male and 4 female specimens of Caucasian ethnicity, without signs of musculoskeletal abnormalities, or previous fracture, or surgery. The mean age of the specimens was 82.8 years (range: 72-99 years). Clinical cadavers were prepared with the Sandeski technique and stored at 4°C. This study was approved by the Horizon Health Network Research Ethics Board.

### Nail Insertion

**Figure 1.**
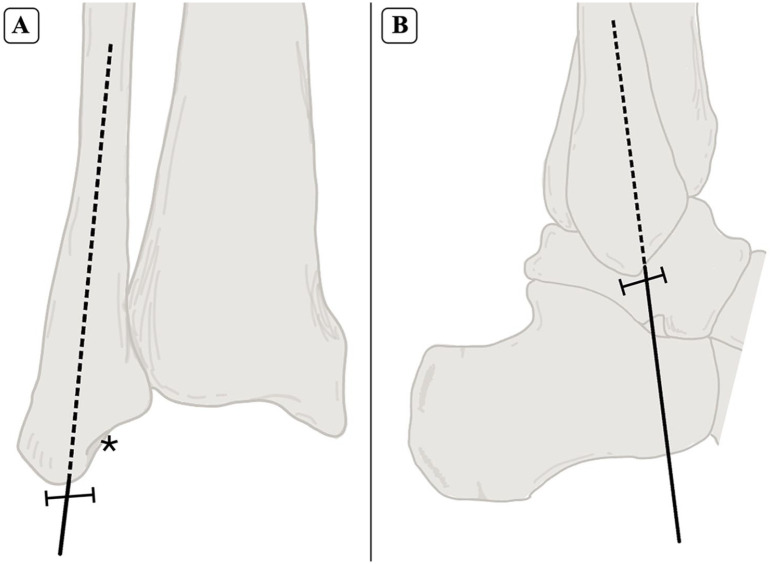
Anterior (A) and lateral (B) views of the fibular start point for intramedullary nailing. The starting point is identified at the tip of the fibula (⊢⊣), positioned lateral to the malleolar fossa (*), with a trajectory aligned to the anatomic axis of the fibula.

**Figure 2.**
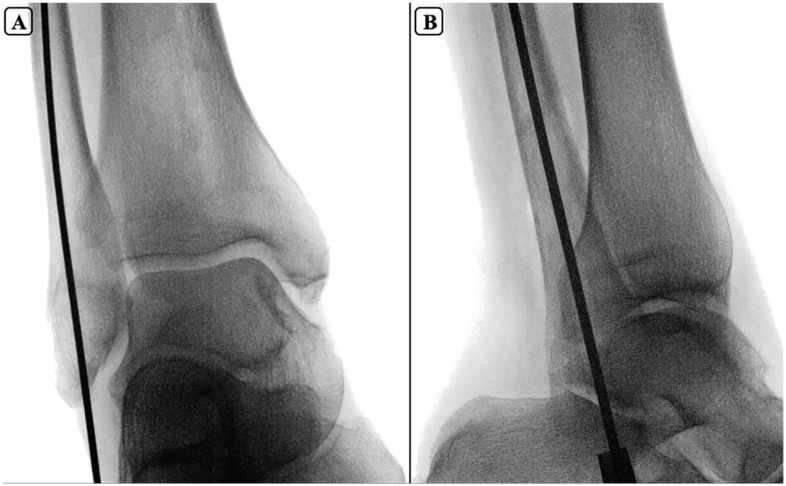
Fluoroscopic mortise (A) and lateral (B) views of guidewires positioned for fibular nail entry, demonstrating alignment with the intramedullary canal.

**Figure 3.**
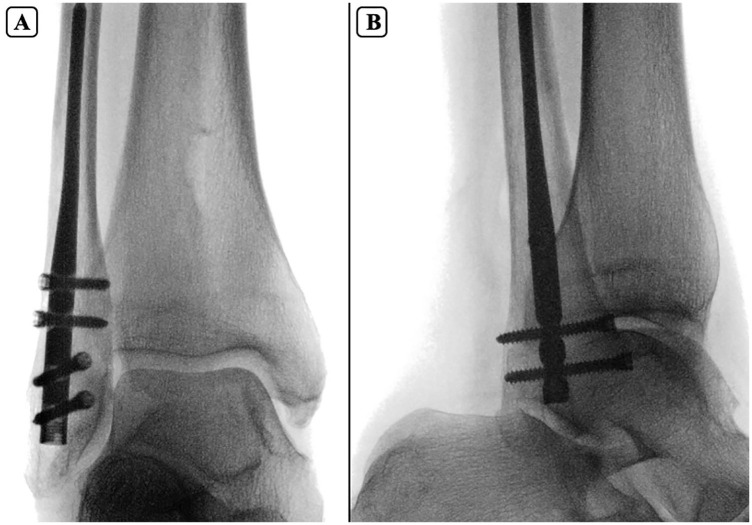
Fluoroscopic mortise (A) and lateral (B) views showing final intramedullary fibular nail placement with 2 anterior-to-posterior and 2 lateral-to-medial locking screws.

A fellowship-trained foot and ankle orthopaedic surgeon performed all the fibular nailing using the procedure described by the manufacturer (Acumed Fibula Nail 2 System). Briefly, a 2-mm percutaneous pin was introduced at the tip of the lateral malleolus, and advanced in line with the fibular intramedullary canal on mortise and lateral projections (Figures 1 and 2). Next, after blunt dissection and insertion of a drill sleeve to protect soft tissue structures around the distal fibula start site, the canal was opened with a 6.5-mm cannulated drill and then sequentially reamed until cortical engagement was noted. The appropriately sized fibular nail (2.6-3.6 mm) was then inserted with 4 distal locking screws. Fluoroscopy was used to ensure adequate placement (Figure 3).

### Dissection and Measurements

An extensile longitudinal approach over the fibula was used to identify the CFL, ATFL, SN, SPN, PL, PB, and the articular cartilage in the lateral gutter. Distances between these structures and the instrumentation were measured with a digital caliper by the surgeon and an anatomist, with the means and SDs calculated for analyses. All measurements were independently performed by both the surgeon and anatomist, with consensus reached on each recorded value during dissection. The mean measurements are reported in Table 1. Although interrater reliability was not formally tested, agreement was achieved in all cases. The shortest distances to the structures were recorded to the nearest 10th of a millimeter, with the SDs and variances reported. Furthermore, the extent of damage to the CFL was quantified. Injury to the CFL was assessed in millimeters, and the percentage of ligament injured was calculated as the width of damaged fibers divided by the total width of the ligament, which was measured slightly distal to the injury site where the ligament remained intact.

**Table 1.** Average Distances (mm) From IM Nail to Nearby Anatomic Structures.

Structure	Minimum	Maximum	Mean	SD
CFL	0.00	2.90	1.20	1.08
ATFL	1.50	8.45	3.43	2.07
SN	9.55	22.65	16.00	4.41
SPN	18.35	48.30	40.21	8.88
PL	1.75	13.15	5.90	3.39
PB	1.25	7.45	3.19	2.03
Cartilage	1.65	7.80	3.45	1.76

Abbreviations: ATFL, anterior talofibular ligament; CFL, calcaneofibular ligament; IM, intramedullary; PB, peroneus brevis; PL, peroneus longus; SN, sural nerve; SPN, superficial peroneal nerve.

### Statistical Analysis

The collected measurements were analyzed using descriptive statistics to determine the frequency of injuries and to calculate the mean, SD, and range of the recorded distances. The observed distances were used to classify risk levels into 3 categories: high (0-5 mm), moderate (5.1-10 mm), and low (over 10 mm); these thresholds have been adopted from prior anatomical literature, but have not been prospectively validated to predict clinical outcomes.^9^

## Results

Average distances and SDs from the fibular nail insertion point to the nearest structures are shown in Table 1.

**Figure 4.**
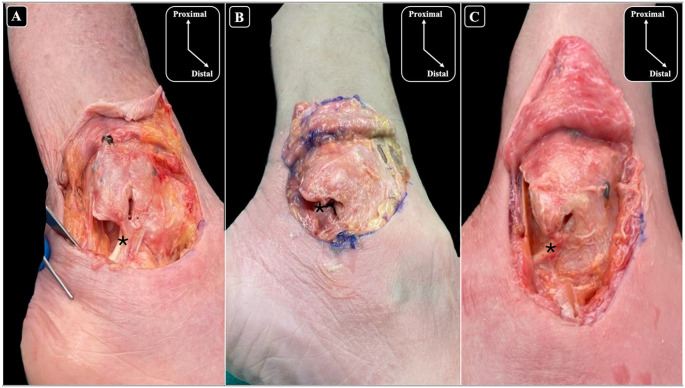
Lateral views of the ankle and anatomical dissections of the fibula showing damage to the calcaneofibular ligament (CFL) caused by nailing, with observed damage percentages of (A) 25%, (B) 14%, and (C) 64%. To facilitate clear visualization of the CFL, the overlying peroneal tendons were removed. The asterisk (*) denotes the location of the CFL.

**Figure 5.**
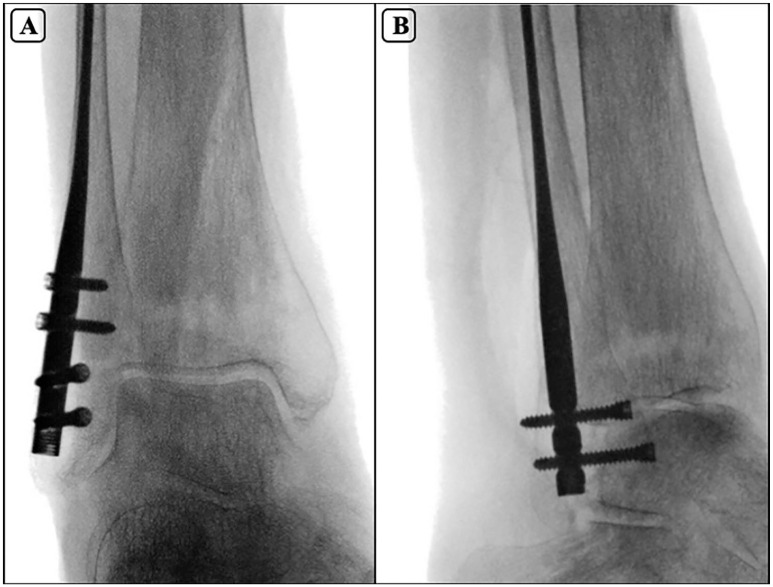
Fluoroscopic mortise (A) and lateral (B) views of the specimen with the greatest calcaneofibular ligament injury (64%), illustrating final fibular nail positioning and relation to adjacent structures.

On average, the distance measured to the CFL (1.20 ± 1.08 mm), PB (3.19 ± 2.03 mm), ATFL (3.43 ± 2.06 mm), and the articular cartilage (3.45 ± 2.07 mm) classified these structures into the high-risk category. The CFL, close to its origin, was the only structure with observed damage in 3 of the 10 cadavers after the nail insertion; the extent of the damage ranged from 14% to 64% of the width of the ligament (Figure 4). To assess for the possibility that a malpositioned start point contributed to iatrogenic ligament damage, the fluoroscopy images for the 3 specimens with CFL damage were scrutinized (Figure 5). Although most of the specimens showed an appropriate start point, 1 ankle had an excessively posterior and laterally positioned start point. For this specimen, establishing the start point was challenging, and it is notable that this specimen sustained the highest degree of damage to the CFL (64%). Nail diameter and length were recorded for each specimen and analyzed in relation to CFL damage; however, no consistent association was observed. Table 2 summarizes the nail dimensions (2.6-3.7 mm diameter; 110-270 mm length) and corresponding CFL injury across specimens.

**Table 2.** Nail Dimensions and CFL Damage Summary.

Specimen No.	Nail Diameter × Length (mm)	CFL Damage?	Damage (%)
1	2.6 × 110	No	-
2	3.0 × 125	No	-
3	2.6 × 145	No	-
4	2.6 × 145	No	-
5	3.7 × 160	No	-
6	2.6 × 190	Yes	25
7	2.6 × 270	Yes	14
8	3.0 × 110	Yes	64
9	3.6 × 190	No	-
10	3.0 × 190	No	-

Abbreviation: CFL, calcaneofibular ligament.

**Figure 6.**
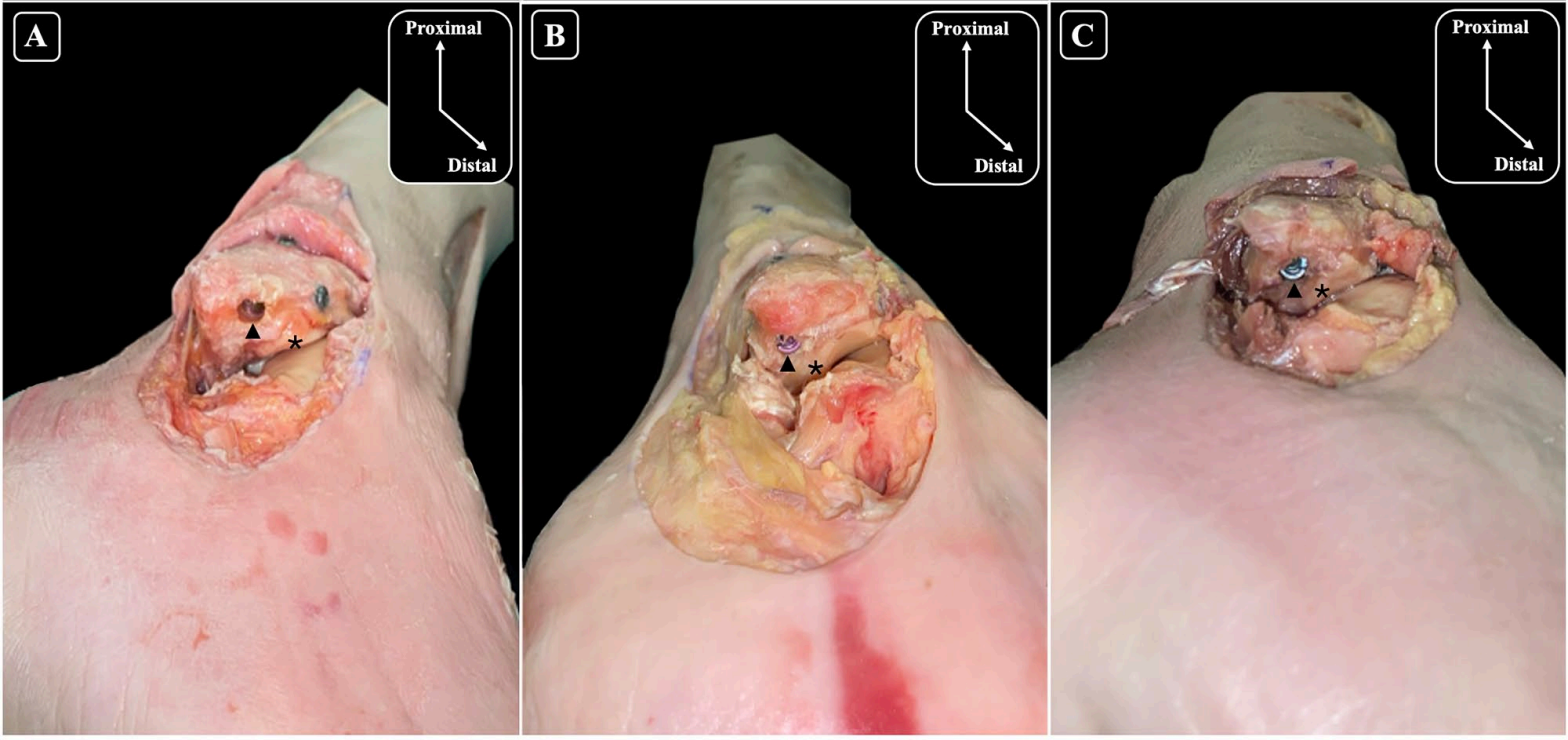
Inferolateral views of the ankle and anatomical dissections of the fibula (A-C) illustrating the relationship between the entry point of the nail and the articular cartilage. The black arrow-head (▲) points to the entry point of the nail, and the asterisk (*) indicates the articular cartilage highlighting its proximity to the nailing entry site across each specimen. Chondral injury was not identified in any of the specimens.

The articular cartilage of the lateral gutter was close to the start point, but no damage was detected. The start point for the nail was posterior and lateral to the cartilage in the lateral gutter in 100% of the specimens (Figure 6).

The PL (5.90 ± 3.39 mm) was in the moderate risk range, and the SN and SPN were low risk. The most proximal anteroposterior locking screw distance to the SPN was also measured to be 33.48 mm (±7.03 mm).

## Discussion

In the surgical management of ankle fractures, fibular nailing is an option that may protect soft tissues, provide stable fixation, and reduce postoperative complications.^1-4,19^ However, the potential for iatrogenic damage with the insertion of the nail through the distal fibular start site has not been adequately characterized. In this study, we found that, overall, the procedure is safe and respectful of the local anatomy. However, even subtle deviations in the entry point can shift the nail trajectory and increase the risk to key structures, such as the CFL.

Our results demonstrated that among the structures evaluated, the CFL demonstrated the highest risk of injury, with damage observed in 3 of 10 specimens. Among the 3 ankles with CFL injury, 2 had partial injuries, while in the third, the damage was more extensive. In that specimen, establishing the start point proved more challenging, resulting in a more posterior and lateral entry that may have contributed to the ligament damage. Although the peroneal tendons are very close and overlie the CFL, no damage was noted to these structures.

The potential for CFL damage with retrograde screw fixation of the fibula was previously assessed. Medda et al^17^ inserted retrograde 3.5-mm cortical screws into a start site 3 mm lateral to the malleolar fossa, which corresponds to the tip of the lateral malleolus. They identified the ATFL (3.33 mm) and CFL (2.66 mm) as the closest structures to the screw head, similar to our findings.^17^ No damage to the ATFL and CFL or the peroneal tendons was identified when proper technique was followed. Telgheder et al^23^ similarly used a starting point at the tip of the lateral malleolus for a 4.5-mm retrograde medullary screw in 10 cadaveric specimens. They found that critical structures (including the sural nerve, peroneal tendons, and ATFL/CFL) remained safely distant, with no iatrogenic injury noted. In this study, we used a starting position on the tip of the fibula, ensuring the starting guidewire stays in line with the intramedullary canal of the fibula on both the mortise and lateral views. Although our starting point was consistent with Medda and Telgheder, an IM fibular nail requires a larger opening drill (6.5 mm in this system) than a screw, potentially contributing to the partial CFL injuries detected in our study.

The clinical consequences of incomplete and isolated CFL damage are not known. Along with the ATFL, the CFL is one of the primary lateral ankle-stabilizing ligaments. The importance of the CFL was previously highlighted in a cadaveric study, where CFL-deficient ankles demonstrated significantly increased lateral ankle instability, alteration in tibiotalar contact mechanics, increased inversion of the talus and calcaneus, and increased medial displacement of the calcaneus.^12^ Whether any of these changes take place in ankles with incomplete injury to the CFL is not known. Interestingly, recent literature about the lateral ankle ligament complex supports the notion that the ATFL is composed of 2 fascicles.^11^ Partial injury to the superior fascicle of the ATFL has been implicated as a contributing factor to subtle ankle instability.^11^ The CFL, at present, is known to be composed of a single band, and whether partial injury can contribute to instability is unknown. Furthermore, multiple studies highlighted the interconnections that exist within the lateral ligament complex, making it possible that damage to a certain part can be compensated by another portion of the unit.^6,11^

Previous investigators examined some of the other pertinent soft tissue structures related to fibular nailing. Goss et al^9^ highlighted the risk to the peroneal tendons and the SPN, emphasizing that these structures were less than 5 mm from the insertion site. In contrast, our findings indicate that the SPN and SN were at low risk. Differences may be due to the variations between the implants used in the studies. The anterior locking screws were the closest to the SPN and the distance between these structures was measured but still fell into the low-risk category (>10 mm). The specific design of the fibular nail and/or the targeting guides for the locking screws may place the SPN in more or less risk, depending on the position of the locking screws. Surgeons need to keep in mind the position of the nerve based on the particular system they are using. Our study also found that the PB was at high risk as it was frequently positioned very close to the nail insertion pathway, nearly resulting in injury.

We found that the articular cartilage was spared in 100% of the specimens in the lateral gutter with a safety margin of 3.45 ± 2.07 mm. By utilizing a start point aligned with the center of the intramedullary canal in both the mortise and lateral views, the nail’s entry points consistently avoided direct contact with the cartilage and nearby structures. The entry points typically positioned the nail laterally and slightly posteriorly away from the articular cartilage. Sparing the articular cartilage is critical to prevent future degenerative changes.^13^

Our study has several weaknesses. A sample size of 10 specimens, although consistent with other cadaveric studies, is relatively small and may limit the broader applicability of our findings. Furthermore, the specimens had a high mean age of 82.8 years and were exclusively of Caucasian ethnicity. These factors limit generalizability and do not capture the anatomical diversity present in the general population.^5^ All specimens also had intact fibulae; in a fresh traumatic fracture, swelling and fragment displacement can alter the soft-tissue relationships around the distal fibula, so the clearances we measured may differ from those encountered intra-operatively. In particular, tissue turgor and hematoma formation in acute fractures may narrow safe zones. Anatomical variability such as leg size and diameter of the medullary canal may influence the proximity of key structures during fibular nailing, and this was not specifically assessed.^8,26^ In addition, although all procedures were performed by a single fellowship-trained surgeon, the potential influence of a learning curve over the course of the 10 procedures cannot be excluded. Finally, we did not assess injury to the lateral malleolar fossa, which is the origin of the posterior talofibular ligament (PTFL).^21,22^ The implications of ATFL and CFL injury on ankle instability have been extensively studied whereas the role of the PTFL remains less clear.^7,12,26^ The susceptibility of the malleolar fossa and PTFL for injury in fibular nailing will need to be assessed in future studies.

## Conclusion

Fibular nailing has been demonstrated to be an overall safe procedure with respect to the surrounding soft tissue structures. Nevertheless, partial CFL injury occurred in 30% of specimens, reinforcing the importance of precise start-point selection. However, these injuries were isolated and typically incomplete. Importantly, although articular cartilage was at high risk, it was spared in all specimens. Additional high-risk structures such as the ATFL and peroneal tendons remained protected when appropriate techniques were employed. Therefore, with careful identification of the ideal start point and adherence to best practices, fibular nailing minimizes the risk of iatrogenic damage and is a safe option in the management of distal fibula fractures.

## Supplemental Material

sj-pdf-1-fai-10.1177_10711007251351314 – Supplemental material for Assessing Risk to Articular Cartilage and the Calcaneofibular Ligament During Fibular Nailing: A Cadaveric Study Supplemental material, sj-pdf-1-fai-10.1177_10711007251351314 for Assessing Risk to Articular Cartilage and the Calcaneofibular Ligament During Fibular Nailing: A Cadaveric Study by Hirbod Abootalebi, William Mayer, Erin Bigney, Siyum Mohiuddin, Xiuming Shi, Madeline Power and Jacob Matz in Foot & Ankle International
